# Efficient Photodegradation of Thiocyanate Ions in Mining Wastewater Using a ZnO-BiOI Heterojunction

**DOI:** 10.3390/ma17153832

**Published:** 2024-08-02

**Authors:** Darlington C. Ashiegbu, David Nkhoesa, Rudolph Erasmus, Herman Johanes Potgieter

**Affiliations:** 1 Sustainable and Innovative Metals and Minerals Extraction Technology (SIMMET) Laboratory, School of Chemical and Metallurgical Engineering, University of the Witwatersrand, Private Bag X3, Wits, Johannesburg 2050, South Africa; david.nkhoesa@students.wits.ac.za (D.N.); herman.potgieter@wits.ac.za (H.J.P.); 2 School of Physics, University of the Witwatersrand, Private Bag X3, Wits, Johannesburg 2050, South Africa; rudolph.erasmus@wits.ac.za

**Keywords:** degradation mechanism, doping, thiocyanate, heterojunction, photocatalysis, synthesis

## Abstract

Mining industries have long relied on cyanidation as the primary method for gold extraction, but this process generates thiocyanates as a problematic byproduct, posing challenges for wastewater treatment and recycling. The stability of thiocyanates makes their reduction or elimination in mining wastewater difficult. In this study, a *p-n* heterojunction of ZnO and BiOI was created and evaluated for its ability to photodegrade thiocyanate ions under simulated solar conditions. Various analytical techniques revealed a highly porous structure with a sponge-like morphology and agglomeration in the synthesized heterojunction. The compound exhibited crystalline patterns without impurity peaks, a slight red shift in absorbance, and Type IV isotherm adsorption. The synthesized heterostructure achieved the complete destruction of thiocyanate ions in less than 30 min. The investigation of different process parameters indicated that the destruction of the contaminant by the heterostructure was influenced by the initial thiocyanate concentration, which decreased as the thiocyanate concentration increased. The peak photodestruction reaction was observed at pH 7. By applying a pseudo-first-order kinetic model, it was found that increasing the catalyst mass to 15 mg raised the rate constant from 0.188 to 0.420 min^−1^, while increasing the pH to 10 led to a 3.5-fold reduction. The strong correlation between the observed data and the predicted values of the pseudo-first-order kinetic model was indicated by the observed (R^2^) values. The findings of this study hold potential significance for mining industries, as it offers a potential solution for eliminating cyanide and thiocyanates from mining wastewater. The elimination of thiocyanate generation in the cyanidation process is crucial for mining companies, making this study valuable for the industry.

## 1. Introduction

Mining industries globally are major contributors to the problem of water pollution because of the production of wastewater in the form of discharged effluents that contain cyanides and thiocyanates. The gold mining industry has relied on cyanidation as the primary leaching method for numerous years [1,2]. From its low-grade ore, gold is dissolved with cyanide to form a complex that is soluble in water. When gold is extracted from ore using cyanide, it can produce hazardous and toxic mixtures of cyanide and thiocyanate as waste products [1].

Thiocyanate forms during the leaching process when cyanide reacts with sulfur species like polysulfide, pyrite, and thiosulfate. Most sulfide minerals, except lead sulfide, can produce thiocyanate, especially under poor aeration and low alkaline conditions [1], where hydrogen sulfide (H_2_S) reacts with cyanide (CN^−^) to form thiocyanate (SCN^−^). Thiocyanate levels vary depending on the sulfur content of the feedstock and can reach significant levels during cyanidation [3]. This formation (Equations (1)–(3)) causes operational issues, including challenges in wastewater discharge and recycling, excessive cyanide consumption, inhibition of gold sorption, and environmental concerns [4]. Despite being less harmful than cyanide, thiocyanate is more stable and difficult to degrade, necessitating processes to reduce its concentration to permissible levels before discharge.

(1) $$S^{0}\;+\;{\mathrm{CN}}^{-}\;\to \;SCN^{-}$$

(2) $$S^{2-}+{CN}^{-}+H_{2}O+1/2O_{2}\to SCN^{-}+2OH^{-}$$

(3) $$S_{2}O_{3}^{2-}+CN^{-}\to SO_{3}^{2-}+SCN^{-}$$

Various methods have been explored for thiocyanate degradation, including biological treatment, physical processes, natural degradation, electrochemical techniques, and oxidation by chlorine and ozone [4]. Biological methods are limited to low concentrations of thiocyanate due to its toxicity [2]. Physical techniques like ion exchange and membrane separation can recover valuable components but have drawbacks such as resin and membrane poisoning, high costs, and limited capacity [2,3,4,5]. Wet oxidation with oxygen can produce hazardous by-products and requires high temperatures, pressure, and expensive equipment [4]. Electrochemical methods are inefficient, chlorine is hazardous, ozone is toxic, and natural degradation is slow [6]. Advanced oxidation processes (AOPs) such as hydrogen peroxide, ozone, and SO_2_/air are effective but expensive and still produce hazardous by-products (Equations (4) and (5)) [3]. Developing novel, efficient methods to degrade thiocyanates into environmentally benign by-products is crucial due to their stability and toxicity.

(4) $$SCN^{-}+3H_{2}O_{2}\to SO_{4}^{2-}+HCN+H^{+}+2H_{2}O$$

(5) $$SCN^{-}+3HSO_{5}^{-}+H_{2}O\to 4SO_{4}^{2-}+HCN+7H^{+}$$

Photocatalysis offers an efficient, eco-friendly method for eliminating thiocyanates, using light energy to promote chemical reactions that degrade pollutants into less harmful products like CO_2_ and H_2_O. Zinc oxide (ZnO) is a popular photocatalyst due to its non-toxicity, low cost, and beneficial properties such as near UV absorption and large excitation binding energy [7,8]. However, ZnO’s wide band gap limits it to UV absorption, and it suffers from rapid charge carrier recombination and photocorrosion in acidic conditions, reducing its photocatalytic efficiency [9]. To enhance ZnO’s performance, researchers suggested methods such as incorporating narrow bandgap semiconductors, doping with metals/non-metals, forming heterojunctions with other semiconductors, and etching onto carbon nanostructures [7,10]. These modifications aim to improve ZnO’s efficiency by reducing recombination rates and expanding light absorption capabilities. 

Bismuth oxyiodide (BiOI) is a *p*-type ternary metal oxide with a small band gap and exceptional corrosion stability. It has excellent photocatalytic activity and very strong visible light absorption. However, one major challenge with using BiOI is the increased recombination of photoinduced charge carriers, which reduces its quantum efficiency. To enhance the photoactivity of BiOI, adequate tailoring is necessary to address the recombination of photoinduced charge carriers and improve its charge migration efficiency [11]. Consequently, BiOI and ZnO are two semiconductors with complementary absorption properties that make them suitable for forming a *p-n* heterojunction for improved photocatalytic performance [7,12]. 

Traditional methods for thiocyanate degradation have not provided sustainable and efficient solutions, and the use of heterojunction photocatalysts like ZnO [10%]-BiOI for this purpose has not been previously studied. Most research on photocatalysis has focused on other contaminants or used single-component photocatalysts, which often suffer from rapid recombination of charge carriers and limited visible light absorption [3]. This study addresses this gap by investigating the photodegradation of thiocyanate ions using a ZnO [10%]-BiOI heterojunction. The research highlights the environmental benefits of this approach, offering a more efficient, cost-effective, and environmentally friendly method for thiocyanate degradation compared to traditional methods. In summary, this work fills a significant knowledge gap in photocatalysis by demonstrating the effectiveness of ZnO [10%]-BiOI heterojunctions in degrading thiocyanate ions, potentially leading to improved methods for treating various environmental pollutants.

In this investigation, a ZnO-[10%]BiOI heterojunction photocatalyst was prepared, characterized, and utilized for the photocatalytic treatment of ferric thiocyanate (SCN^−^) in aqueous media. The synthesized heterostructure was additionally applied to investigate the influence of some operational parameters. This study builds on previous research that optimized the weight percentage doping of BiOI into the ZnO lattice, which was applied for the degradation of 2-chlorobiphenyl [7]. The findings from this study are useful in wastewater treatment applications, and suggest that the ZnO-[10%]BiOI heterostructure is an efficient photocatalyst and can be applied in the destruction of thiocyanates, specifically in the mining industry. To the best of the knowledge of the authors, the development of very photoactive and effective heterojunction photocatalysts with the capacity for quick destruction of thiocyanates in aqueous media has not been reported previously. In addition to the current investigation, the authors plan to perform a mineralization study, radical scavenger studies, and ascertain the catalyst stability in future work.

## 2. Experiment

### 2.1. Materials

Oxalic acid, bismuth nitrate pentahydrate ((Bi(NO_3_)_3_·5H_2_O)), and potassium iodide (KI) were acquired from Sigma-Aldrich (St. Louis, MO, USA). Zinc acetate dehydrate (ZAD; [Zn(CH_3_COO)_2_·2H_2_O], ethanol, methanol, potassium thiocyanate (KSCN) and ferric chloride (FeCl_3_) were acquired from Associated Chemical Enterprises (ACE) (Johannesburg, South Africa). Hydrochloric acid (HCl) and ammonium hydroxide (NH_4_OH) were obtained from the School of Chemical and Metallurgical Engineering laboratories at the University of the Witwatersrand. All sample preparations utilized deionized water that had undergone purification using a Millipore system (Hach, Johannesburg, South Africa). As all of the chemicals and solvents used in the tests were of AR (analytical reagent) grade, no additional purification was performed on them before use. Ferric chloride and potassium thiocyanate were each dissolved in 100 mL of deionized water and reacted to form a red complex of ferric-thiocyanate (Fe(SCN)_3_) stock solution, which acted as a model solution for the work. From this stock solution, a working concentration of 40 ppm and other concentrations were prepared. 

### 2.2. Synthesis of ZnO

A sol-gel method was used in the synthesis of ZnO nanoparticles. ZAD [Zn(CH_3_COO)_2_·2H_2_O)] was dissolved in ethanol and oxalic acid was gradually added into the solution. This was followed by a 30 min reflux of the solution at 60 °C, which resulted in gel formation as refluxing was extended for 60 min at 50 °C. The resulting xerogel was allowed to cool and then dried in an oven at 80 °C. Finally, the sample was calcined at 500 °C to produce ZnO.

### 2.3. Synthesis of ZnO-[10%]BiOI

**Figure 1:**
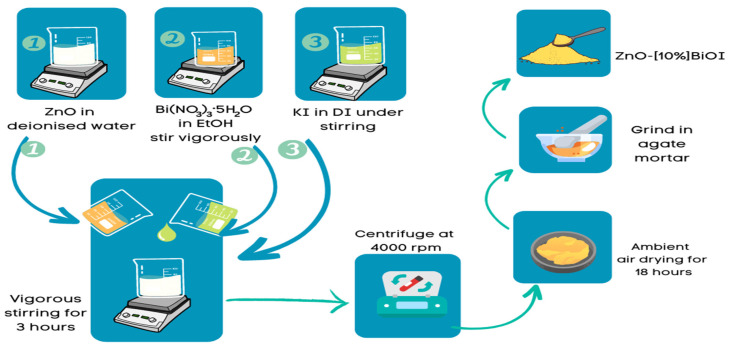
Synthesis of ZnO-[10%]BiOI heterojunction.

As reported elsewhere, the ZnO-[X]BiOI heterostructure was synthesized by suspending ZnO in deionized water. Bismuth nitrate pentahydrate [Bi(NO_3_)_3_·5H_2_O] (approximately 11 g) was separately added to ethanol and stirred vigorously [7]. The Ethanol-Bi(NO_3_)_3_·5H_2_O solution was then added to the ZnO suspension, and stirring continued. Under intense and continuous stirring, deionized water was utilized to dissolve potassium iodide (KI, approximately 5 g), which was then gradually added to the mixed liquor. After 3 h of rigorous stirring, the suspension was centrifuged to segregate the solid product from the liquid phase. The solid product was subsequently air-dried. Once dried, the product was collected and ground in an agate mortar to obtain the ZnO-[10%]BiOI heterojunction compound. The synthesis process is shown schematically in Figure 1.

### 2.4. Characterization

A Carl Zeiss Sigma FE-SEM equipped with an Oxford X-act EDS instrument (Oxford Instruments, Oxfordshire, UK) was used to determine the surface morphologies and elemental analyses of the materials, a UV 1800 Shimadzu UV-Vis Spectrophotometer (Shimadzu Corporation, Kyoto, Japan) was used for all of the optical experiments, a Bruker D2 XRD instrument (Bruker Corporation, Billerica, MA, USA) was used to measure the diffraction patterns of the composites, and a Micrometrics TriStar 3000 instrument (Micrometrics Instrument, Norcross, GA, USA) was used to measure BET surface area and the corresponding adsorption isotherms. The Debye-Scherrer relation was used to calculate the mean crystallite sizes of the fabricated composites via the most intense peaks from the XRD pattern, and Tauc plots obtained from UV-Vis spectra were employed to ascertain the band gap measurements. During the synthesis, some liquid-suspended particles were separated using a Hettich ROTOFIX Benchtop Centrifuge (Andreas Hettich GmbH & Co. KG, Tuttlingen, Germany). All weighing was performed using Radwag Wagi Elektroniczne electronic balances (Radwag Wagi Elektroniczne, Radom, Poland), and all pH values were measured using an OHAUS Starter 3100 pH meter (OHAUS Corporation, Parsippany, NJ, USA). An AM 1.5 G 100 mW/cm^2^ (300–1100 nm) lamp (Newport Corporation, Irvine, CA, USA) was used as the solar simulator for this study. 

### 2.5. Photocatalytic Protocol

The degradation of thiocyanate (SCN^−^) by sunlight was used to study and ascertain the photocatalytic performance of the fabricated ZnO-[10%]BiOI heterojunction photocatalyst. In a typical experiment, 100 mL of Fe(SCN)_3_ complex with a working concentration of 40 ppm was prepared in a 250 mL beaker, placed on a stirrer plate, and positioned 30 cm away from the solar simulator. Prior to this, a 3 mL aliquot of thiocyanate was poured into a cuvette and analyzed for initial absorbance using a UV-vis spectrophotometer. The appropriate dose of heterojunction catalyst was subsequently added and magnetically stirred in the dark to allow an adsorption-desorption equilibrium to be established. To ensure that the photocatalysts were properly dispersed, the suspension was continuously stirred. To adjust the pH in all the experiments, hydrochloric acid (HCl) and ammonium hydroxide (NH_4_OH) were employed. The solution was covered with a box to prevent any external light sources from interfering with the process and the solar simulator was switched on. In order to prevent any radiation from escaping during the photocatalytic experiments, the beaker’s surface was covered. A 5 mg sample of the heterojunction catalyst was added while stirring the reaction mixture. Aliquots of the reaction mixture were withdrawn at 3 min intervals, using a syringe attached to a 0.22 µm filter (to filter off the catalyst) and analyzed on a UV-vis spectrophotometer at the peak absorbance wavelength of 460 nm [1,13]. 

## 3. Results and Discussion

### 3.1. Characterization of ZnO-[10%]BiOI Heterojunction

**Figure 2:**
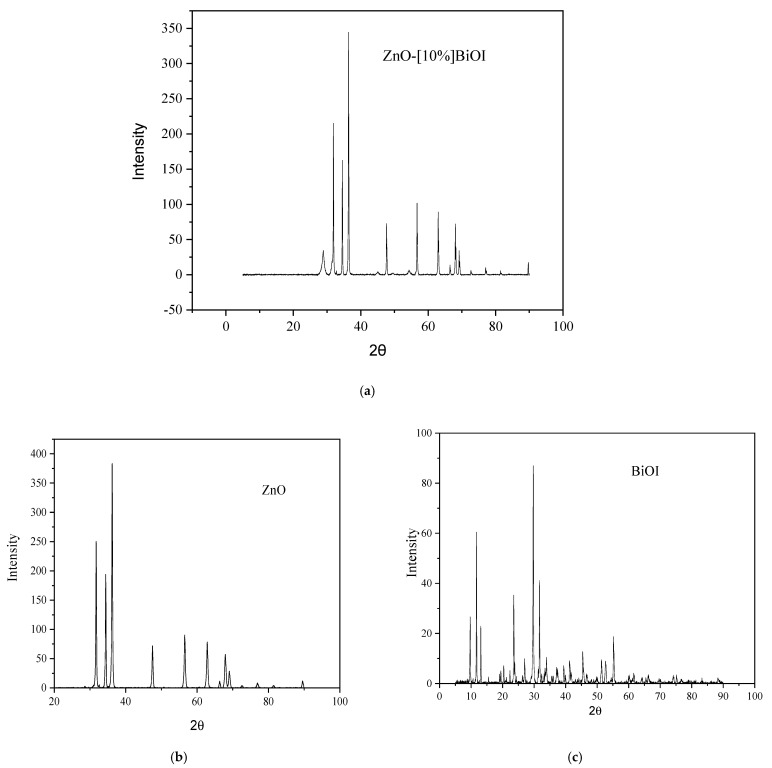
(**a**) XRD pattern of ZnO-[10%]BiOI (**b**) XRD pattern of pristine ZnO (**c**) XRD pattern of pristine BiOI.

As reported previously, the X-ray diffraction pattern of the heterojunction showed distinct and sharp peaks at 2θ values of 31.7°, 34.4°, 36.1°, 47.3°, 56.3°, 62.6°, 66.3°, 67.9°, and 69.1°, corresponding to the 100, 002, 101, 102, 110, 103, 200, 112, and 201 crystal planes of hexagonal ZnO [7]. The peak observed at 29° is ascribed to the 102-diffraction plane of BiOI (see Figure 2). The heterojunction’s diffraction peaks are narrow and intense, indicating a high degree of crystallinity. The presence of other smaller peaks of BiOI is also identified, as presented in Figure 2. The XRD patterns show excellent agreement with the tetragonal BiOI (Figure 2c) (JCPDS card 00-010-0445) and hexagonal ZnO (Figure 2b) (JCPDS card 301-075-6445). The peaks exhibit characteristic features of both pristine BiOI and ZnO crystalline phases. Notably, no impurity peaks were detected, affirming the heterojunction’s purity, good synthesis, and the perfect dispersion of BiOI on the ZnO nanoparticle surfaces.

Using the Debye-Scherrer equation, the mean particle sizes of the prepared semiconductor composites were determined from the Full Width at Half Maximum (FWHM) extracted from the diffraction peaks, as shown below:

(6) D=K[(λ/(βcosƟ)]

where *λ* = 1.54056 nm is the diffraction wavelength, θ is the Bragg diffraction angle of the XRD peak, and β is the measured broadening diffraction line peak at an angle 2θ at half of its highest intensity (in radian). 

The average particle sizes of pure ZnO, BiOI and ZnO-[10%]BiOI were determined as 37.6, 40 and 31 nm, respectively. When compared to pure ZnO, the mean crystallite size of the heterojunction decreased with the doping of BiOI into the ZnO lattice. The tetragonal BiOI may have infiltrated the ZnO hexagonal wurtzite crystal structure, causing a crystal growth-induced collapse, which explains the variation in the heterojunction’s crystallite size when compared to ZnO.

**Figure 3:**
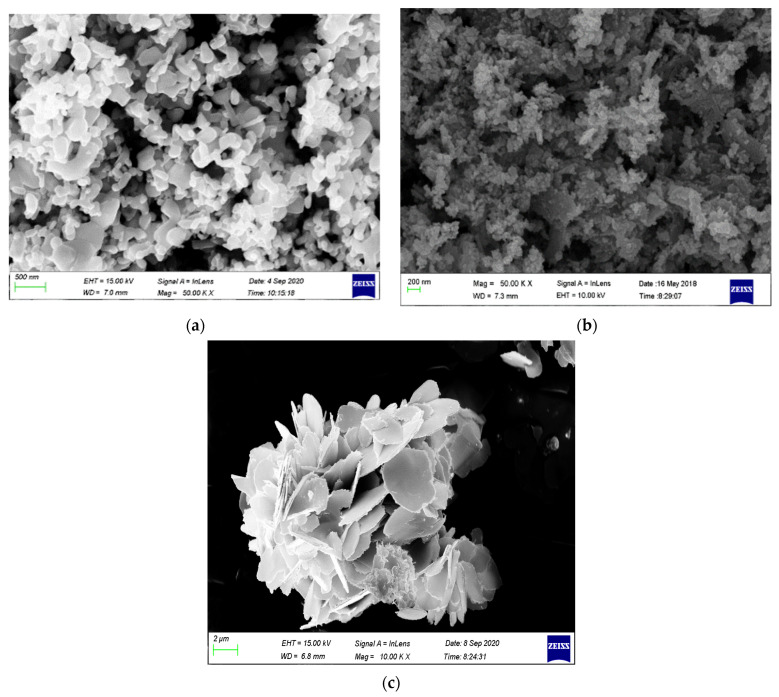
SEM images of (**a**) ZnO-[10%]BiOI heterojunction (**b**) ZnO (**c**) BiOI.

A FE-SEM was utilized to study the morphology of the ZnO-[10%]BiOI heterojunction. Figure 3a illustrates that the surfaces of the heterojunction are non-uniform with agglomeration and lack a specific orientation. In contrast, Figure 3b shows that pure ZnO samples obtained at 500 °C exhibit a sponge-like morphology, with high porosity and aggregation caused by the chemical reactions at high temperatures. This caused a thick morphology due to the complete decomposition of the organometallic precursor. Scanning Electron Microscopy (SEM) of the BiOI catalyst revealed an irregular, plate-like morphology, as depicted in Figure 3c. The solvents used during synthesis, in combination with the hydrothermal method, likely influenced this morphology. Specifically, the low-viscosity solvents contributed to the plate-like structure by facilitating a high diffusion rate of ions.

Based on these findings, it can be deduced that the morphology of the heterojunction is heavily influenced by the BiOI loading in ZnO.

The results of the energy dispersive X-ray spectroscopy (EDS) analysis indicate that the composites contain only the expected elements and appropriate stoichiometry. Supplementary Information Figure S1a,b presents the EDS spectra of the composites.

The absorption properties of the composites were determined using a UV-Vis spectrophotometer. The spectra were recorded within the 200–900 nm range at room temperature. The bandgaps of the composites were derived by deducing the linear portion of the plot of **(*αhν*)^2^** vs. photon energy, using Tauc’s plots via the Kubelka-Munk equation (Equation (7)) as presented below:

(7) $$\alpha hv=A{\left(hv-E_{g}\right)}^{n}$$

where ***α*** represents the absorption coefficient, ***A*** is a constant, ***h*** is Plank’s constant, ***ν*** is the photon frequency, ***E_g_*** is the band gap, and ***n*** is equal to 1/2 or 2 for the transition being direct or indirect, respectively. Extrapolating the linear region in a plot of **(*αhν*)^2^** against ***hv*** gives the band gap values. 

The absorption spectra and Tauc plots of the composites are shown in Supplementary Information Figure S2a–c. The absorption spectra of ZnO and ZnO-[10%]BiOI composite were identified at 373 and 378 nm, respectively. The incorporation of BiOI into ZnO caused a slight red shift in the absorption spectra of the heterostructure. Some other studies reported similar observations [11,12].

The bandgap of the heterojunction was estimated at 3.0 eV while the characteristic bandgap of ZnO was measured at 3.37 eV. Doping ZnO with BiOI caused bandgap narrowing, which corresponds to the observed slight red shift in the absorption spectra of the heterojunction compound.

The nitrogen (N_2_) adsorption-desorption isotherm and Barret-Joyner-Halender (BJH) pore distribution curves were utilized to ascertain the surface area and textural properties of the photocatalysts. The obtained results from the N_2_ isotherm analysis were juxtaposed with the IUPAC classification. The ZnO-[10%]BiOI heterostructure’s specific surface area was measured as 19.79 m^2^/g, which is significantly larger than the surface area of pure ZnO and BiOI, which were measured as 1.93 m^2^/g and 2.61 m^2^/g. The incorporation of BiOI into ZnO caused an increase in the surface area when compared to pure ZnO [14]. 

**Figure 4:**
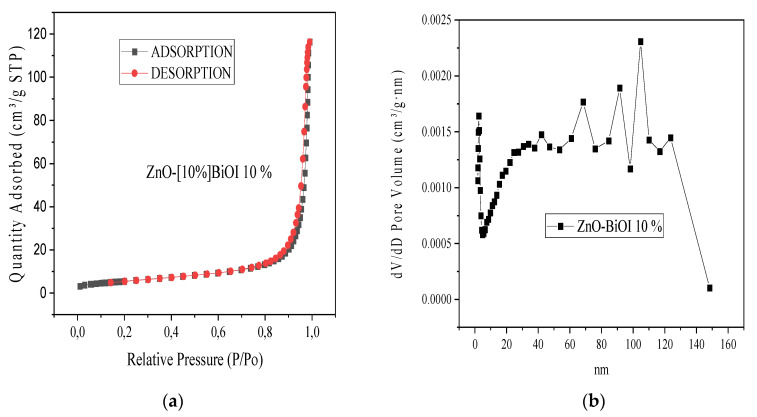

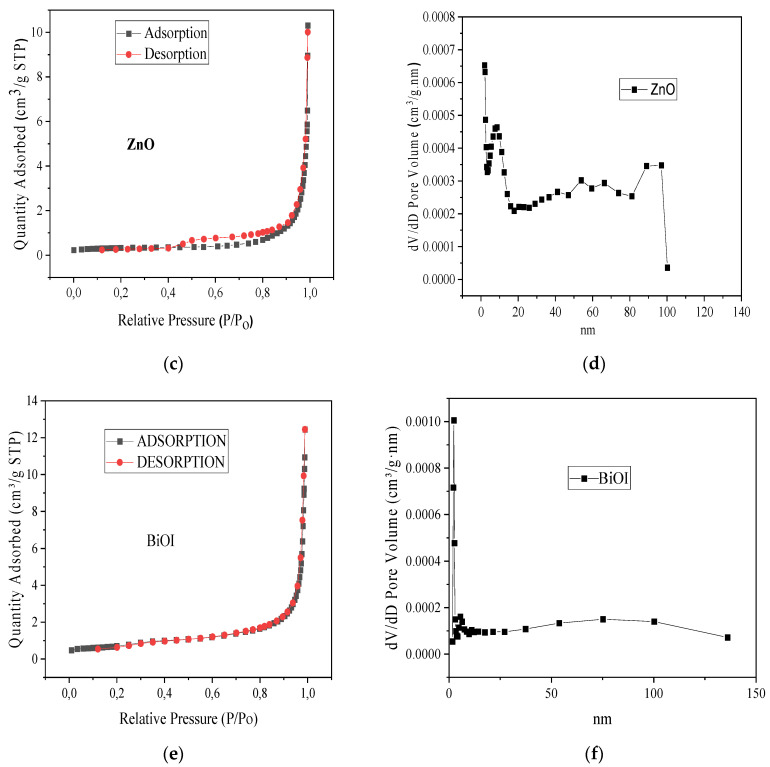
Textural properties (**a**) N_2_ adsorption-desorption isotherm of ZnO-[10%]BiOI (**b**) Barret-Joyner-Halender (BJH) pore size distribution of ZnO-[10%]BiOI (**c**) N_2_ adsorption-desorption isotherm of pristine ZnO (**d**) Barret-Joyner-Halender (BJH) pore size distribution of ZnO (**e**) N_2_ adsorption-desorption isotherm of BiOI (**f**) Barret-Joyner-Halender (BJH) pore size distribution of BiOI.

The N_2_ adsorption-desorption isotherm of the heterostructure conforms to a Type IV isotherm and does not exhibit any hysteresis, as shown in Figure 4a. The BJH curve indicates a mix of mesopores and macropores, with a higher presence of macropores (Figure 4b). For the ZnO sample, the N_2_ adsorption-desorption isotherm (Figure 4c) exhibits a Type V isotherm with H_III_ hysteresis. The BiOI sample displays characteristics of a Type IV isotherm, indicating a mesoporous structure with some macropore distribution (Figure 4e). The corresponding Barrett-Joyner-Halenda (BJH) pore diameter distribution plot for the BiOI material (Figure 4f) confirms the variable pore diameter distribution.

### 3.2. Photocatalytic Degradation Evaluation

**Figure 5:**
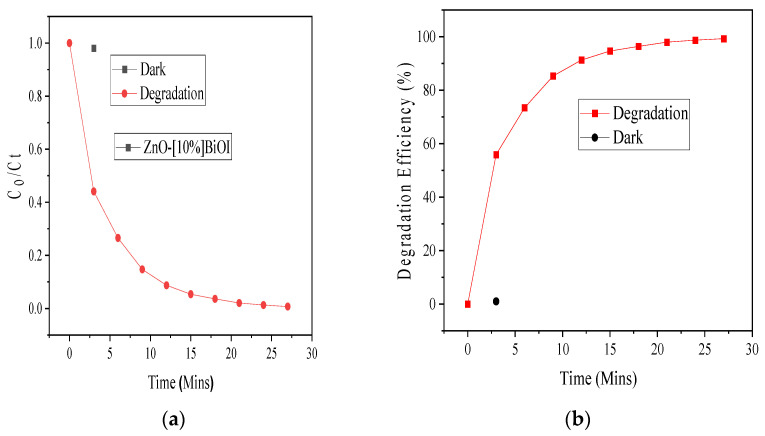
(**a**) Photodegradation of 40 ppm SCN^−^ by 5 mg ZnO-[10%]BiOI (**b**) photodegradation efficiency.

The destruction of thiocyanate under solar radiation simulation was utilized to evaluate the efficiency of the ZnO-[10%]BiOI heterostructure. Figure 5a,b show the photodegradation plots and destruction efficiencies of 40 ppm thiocyanate by 5 mg of the as-prepared heterojunction at its peak absorption wavelength of 460 nm. Thiocyanate was totally eliminated in less than 30 min by the synthesized ZnO-[10%]BiOI heterojunction. The work by Mediavilla and colleagues (2019) reported that nearly 4 h was necessary for the elimination of 50% of the thiocyanates through the application of commercial titanium dioxide (TiO_2_) [3]. This highlights the efficiency and improved photocatalytic activity of the fabricated heterojunction over bare and undoped photocatalysts. The efficient and quick destruction of SCN^−^ by the ZnO-[10%]BiOI heterostructure is attributed to the inhibition of recombination of the charge carriers due to the *p-n* junction between ZnO and BiOI, as well as increased surface area, which provided more catalytic sites that allowed more electrons and holes to participate in the reaction, increased light absorption enabled by the visible light properties of BiOI, and reduced bandgap and generation of additional hydroxyl radicals to enhance the photocatalytic active sites [7,15]. 

### 3.3. Effect of Initial Photocatalyst Loading

**Figure 6:**
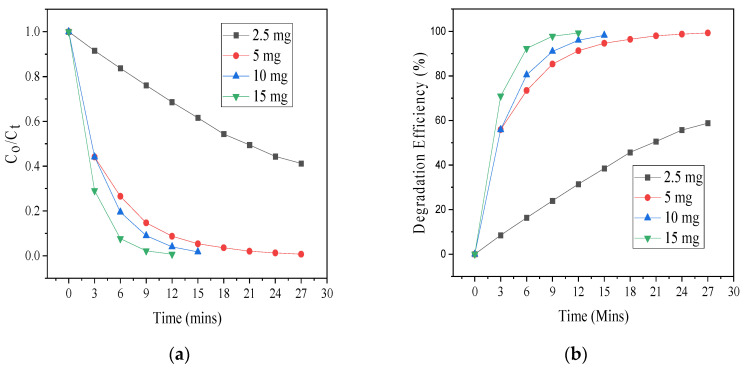
(**a**) Photodegradation of 40 ppm SCN^−^ at varying initial ZnO-[10%]BiOI dosage (**b**) photodegradation efficiencies.

To understand the influence of catalyst dose in the destruction of SCN^−^, the dosage of the heterojunction was varied from 2.5 to 15 mg, while the concentration of thiocyanate was kept constant (40 ppm). Figure 6a,b show the photodestruction of 40 ppm thiocyanate at varying initial ZnO-[10%]BiOI loadings and the corresponding degradation efficiencies. From the plots, it can be deduced that only approximately 60% degradation of SCN^−^ was achieved with 2.5 mg of catalyst mass in 27 min, while 100% destruction was achieved with 10 mg of catalyst dose in 15 min. The highest efficiency was observed when 15 mg of the heterojunction was applied to completely destroy the thiocyanate in 12 min. The degradation efficiency is clearly affected by initial catalyst mass, as was also reported by other authors [16,17]. This is because at low catalyst doses, there is a limited presence of active sites [18]. The higher photocatalytic activity observed at increased catalyst doses in this study is ascribed to the presence of more reaction sites on the heterojunction’s surface for the participation of more electrons and holes in the reaction [19,20].

### 3.4. Effect of Initial SCN^−^ Concentration

**Figure 7:**
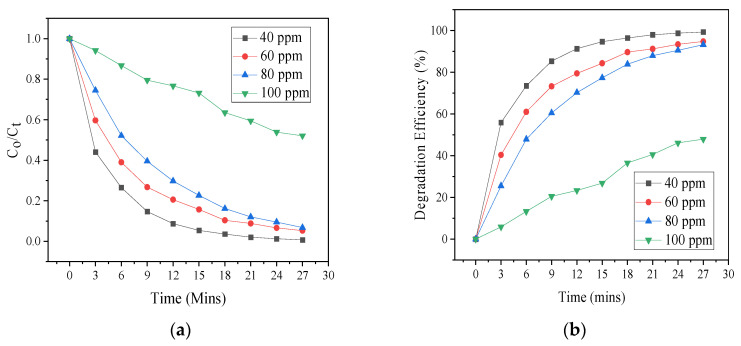
(**a**) Photodegradation of SCN^−^ at varying concentration (**b**) degradation efficiencies.

The destruction of SCN^−^ at varying initial concentrations (40, 60, 80, and 100 ppm) with a catalyst dose of 5 mg was studied. As presented in Figure 7, the highest destruction (100%) was observed at 40 ppm, while the lowest degradation was observed at 100 ppm (approximately 45%) after a reaction time of 27 min. At 80 ppm, approximately 90% degradation was achieved, while 93% degradation was achieved at 60 ppm SCN^−^ concentration. The destruction efficiency reduced with increasing the initial concentration of SCN^−^. A crucial influence that hinders photodegradation at elevated concentrations is the light screening effect caused by particles in the solution [21]. At high concentrations, there is inhibition of active sites which reduces destruction efficiency [22,23]. The observed reduction in the destruction of SCN^−^ by ZnO-[10%]BiOI at an increased pollutant concentration is attributed to the inhibition of the photocatalyst by thiocyanate molecules adhering unto the photocatalyst surface. This means that degradation is inhibited because the number of catalytic sites and concentration of OH· are overwhelmed by the pollutant. As a result, at high concentrations the ratio of reactive radicals to pollutant molecules is reduced and the degradation efficiency is decreased [24,25,26]. The work by Zargaran and colleagues (2020) reported similar findings [27].

### 3.5. pH Effect

**Figure 8:**
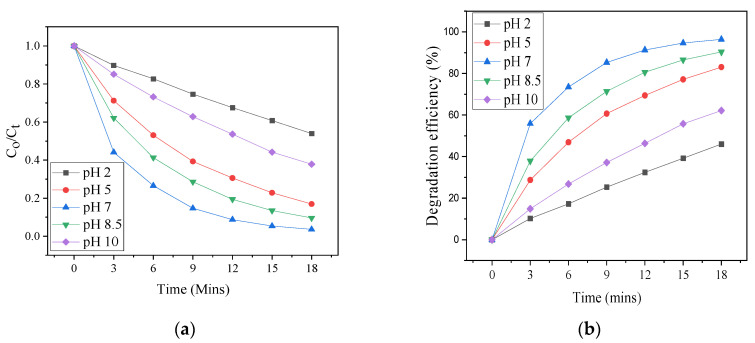
(**a**) Photodegradation of SCN^−^ at varying pH (**b**) degradation efficiencies.

The influence of pH on the destruction of SCN^−^ was investigated in the pH range of 2–10 with a 5 mg catalyst dose and 40 ppm pollutant concentration. Figure 8a,b show the photodestruction of SCN^−^ under varying pH conditions. As shown in Figure 8b, the lowest photo-activities of 45% and 60% were observed at very acidic pH (2) and high alkaline pH (10), while the optimum activity of 100% was obtained at a pH value of 7. Variations in the pH of a solution can alter photocatalytic reactions and affect the rates of adsorption-desorption because of the photocatalyst’s surface charge, which may affect the performance [22,27]. As already established, the point of zero charge (pH_pzc_) of BiOI is around a pH of approximately 3, while the PZC of ZnO is at approximately pH = 9 [28,29,30]. The optimum photocatalytic performance was achieved at pH 7, which is the measured pH of the SCN^−^ solution in this study. The work by Huang and co-workers obtained a similar outcome [31]. The low activity of the heterostructure at alkaline pH is ascribed to coulombic repulsion between the surface of the catalyst and the hydroxide anions (OH^−^), which may have inhibited photo-oxidation [20,32]. The poor performance of the heterostructure in an acidic medium is because at low pH, ZnO has a propensity to aggregate, which reduces pollutant adsorption and photon absorption. Due to the anchored oxide’s strong agglomeration, there may not be enough active sites for reactions to take place [33].

### 3.6. Kinetic Study

**Figure 9:**
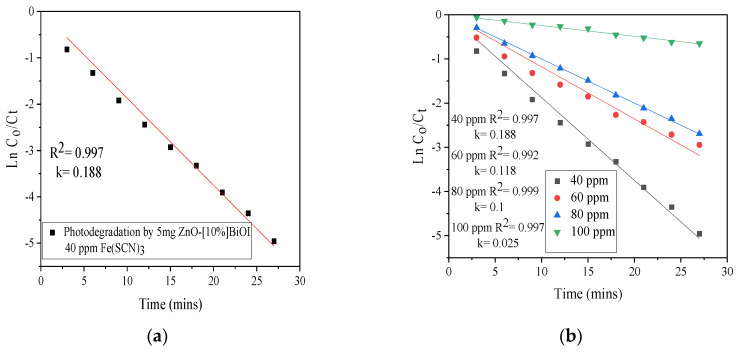

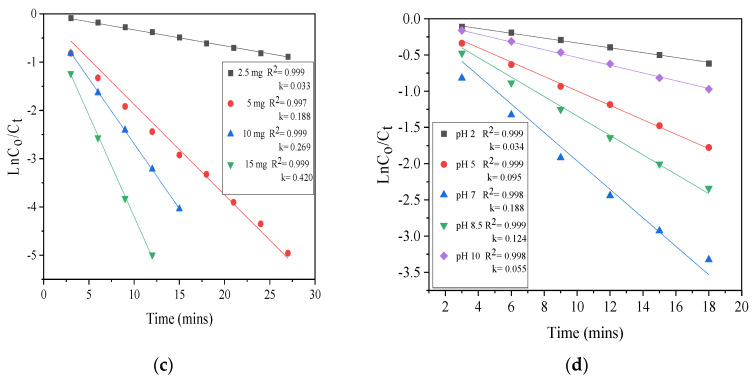
Kinetic fits of (**a**) photodestruction of SCN^−^ by 5 mg ZnO-[10%]BiOI (**b**) effect of initial SCN^−^ concentration (**c**) effect of photocatalyst dose (**d**) pH effect.

The pseudo-first-order kinetic model was observed to best describe the photodestruction of SCN^−^. From the linear plot (Figure 9a), it was found that the photodegradation of SCN^−^ by 5 mg of ZnO-[10%]BiOI could be fitted to the pseudo-first-order reaction kinetic model. The rate constant of the reaction was observed to be 0.188 min^−1^. The kinetic data indicates that when the heterostructure is applied, it takes 4 min for the SCN^−^ concentration to be halved to its initial concentration. Five initial pH values (2, 5, 7, 8.5, and 10, respectively) for 40 ppm of SCN^−^ solution were used to study their respective influences in the photodestruction reaction. After performing a linear regression between the natural logarithm of the ratio of the pollutant concentration at time *t* to its initial concentration [*ln(C/C_o_)*] and the experimental reaction time, it was found that all pH values fit the pseudo-first-order reaction kinetic model in the destruction of SCN^−^. Increasing the pH from 7 to 10 caused a 3.5-fold reduction in the rate constant (0.188–0.055 min^−1^), while reducing the pH to 2 led to an over 5-fold reduction in the rate constant (0.188–0.033 min^−1^), as shown in Figure 9d. At varied catalyst loadings (Figure 9c), it was observed that increasing the photocatalyst dose from 5 to 15 mg caused an increase in the rate constant values (0.188–0.420 min^−1^). At varied SCN^−^ concentrations, the degree of SCN^−^ destruction was affected by its initial concentration, and decreased as the concentration increased, as presented in Figure 9b. The high R^2^ values indicate a strong correlation between the observed data and the predicted values of the pseudo-first-order kinetic model. 

### 3.7. Photoluminescence Study

Steady-state photoluminescence (PL) spectra were acquired at room temperature using a Horiba QM8000 spectrofluorometer (Horiba, Kyoto, Japan) with a Xe lamp as an excitation source and a 320 nm long-pass filter to suppress the higher order reflections of the excitation line at 300nm. The emission spectra are intensity-corrected for the emission spectrograph throughput and detector efficiency.

**Figure 10:**
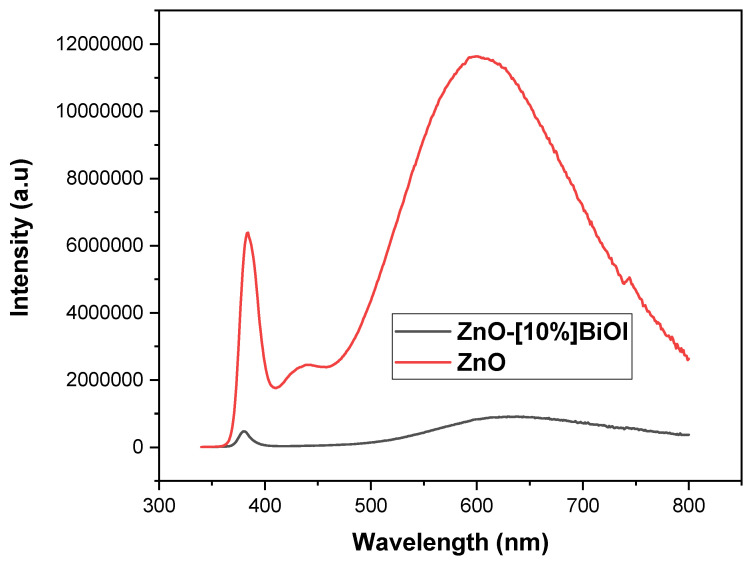
Photoluminescence spectra of the composites.

Under exactly the same measurement conditions, the PL from the 10%BiOI-doped sample is about 10 times less than the PL from the undoped ZnO starting material. This reduction in PL intensity implies that for the BiOI doped sample, the valence electrons excited by the incident light at 300 nm are taking part in non-radiative processes, such as electrochemical or catalytic reactions, rather than undergoing the transition back to the valence band with associated photon emission at 380 nm (bandgap energy at room temperature). This also confirms that a charge transfer occurred from the BiOI layer to the ZnO in the heterojunction, which causes a reduction in electron-hole recombination and illustrates an enhanced photoinduced charge separation. The fairly narrow peak at 380 nm presented in Figure 10 is the band-to-band luminescence transition corresponding to the bandgap. For the 10% BiOI loaded ZnO sample, there is a broad orange-red emission peak with a maximum of around 630 nm. For the ZnO, there is a weaker violet band centered at 442 nm and then a broad orange-red band centered at approximately 600 nm. The broad orange-red bands have been associated with various intrinsic defects in ZnO, such as the positive charge state of the oxygen vacancy. 

### 3.8. Proposed Photodestruction Mechanism

**Figure 11:**
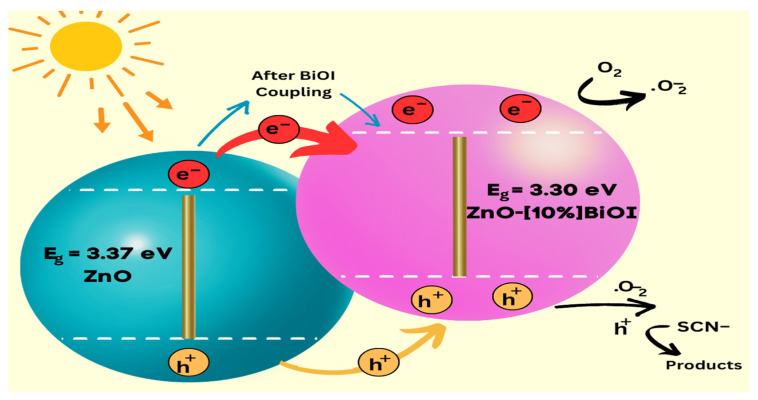
Suggested ZnO-BiOI charge transfer mechanism.

The fabrication of a ZnO-BiOI heterostructure yielded an enhanced photocatalytic activity [34,35,36]. Doping semiconductor photocatalysts with varying crystal structures and band orientation lead to the efficient utilization of incident photons, and ultimately the charge separation is made efficient due to the presence of an electric field interface [7,36], as shown in Figure 11. ZnO has its Fermi level around the *CB*, while the Fermi level of BiOI is near the *VB* [37,38]. A realignment of the Fermi levels of both semiconductors is achieved when ZnO is doped with BiOI, and a reduction in the resistance of surface carrier migration occurs [39]. The narrow bandgap BiOI is excited by visible light, and its valence band electrons transfer to its conduction band. An internal electric field drives the photogenerated electrons in the conduction band of BiOI to ZnO, while the h^+^ are left on the valence band of the BiOI. The effective charge carrier generation/separation and their subsequent inhibition from recombination effectively caused the improved activity of the ZnO-[10%]BIOI heterojunction [12,40]. The photodegradation mechanism proposed from the experimental results is as follows:

(8) $$BiOI\;\overset{hv\left(\lambda \geq visible\;light\right)}{\to }\;\;BiOI\left({h_{VB}}^{+}+e_{CB}^{-}\right)$$

(9) $$BiOI\left(e_{CB}^{-}\right)\;\overset{migration}{\to }ZnO\left(e_{CB}^{-}\right)$$

(10) $$ZnO\left(e_{CB}^{-}\right)+O_{2}\;\to \;ZnO+O_{2}^{.-}$$

(11) $$O_{2}^{.-}+H^{+}\to \;\dot{OH}$$

(12) $$O_{2}^{.-}and\;\dot{\dot{OH}+{Fe(SCN)}_{3}\;\;\to Photodestruction(products)}$$

### 3.9. Chemical State Analysis

**Figure 12:**
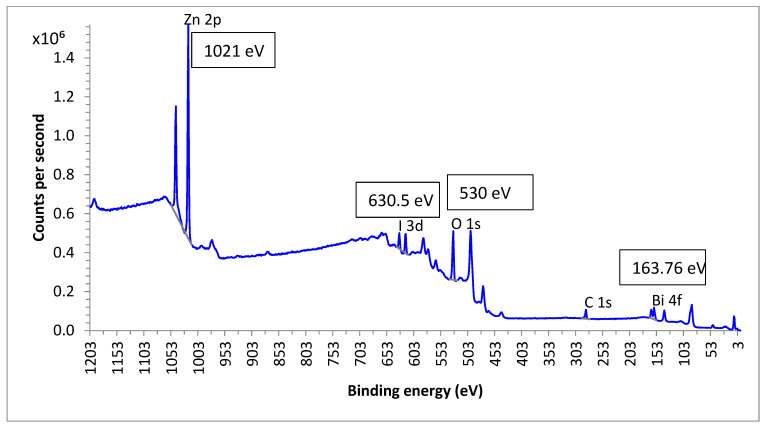
XPS spectra of the ZnO-[10%]BiOI heterojunction.

The examination of oxidation states and surface chemical compositions was conducted using X-ray Photoelectron Spectroscopy (XPS). In Figure 12, the XPS spectrum of the synthesized heterojunction is presented, featuring peaks corresponding to Zn (2p), Bi (4f), O (1s), and I (3d) elements, along with a discernible peak for carbon, reflecting its presence in the instrument. The two peaks observed at 158.46 eVand 163.76 eV are attributed to Bi 4f_5/2_ and Bi 4f_3/2_, respectively, with an energy difference of 5.3 eV, indicating the predominant valence state of the Bi element in the heterojunction as +3. Notably, the intense peaks at 1021 eV and 1044 eV are identified as Zn 2p_1/2_ and Zn 2p_3/2_, respectively, with a peak separation of 23 eV, signifying the presence of Zn^2+^ cations in the ZnO-BiOI heterostructure. The peaks at 619 eV and 630.5 eV correspond to I 3d_5/2_ and I 3d_3/2_, respectively, arising from the spin-orbit splitting of I 3d_5/2_ and I 3d_3/2_. The peak difference of 11.5 eV indicates the presence of I^−^ anions. The O 1s spectrum reveals a single type of lattice oxygen in the heterojunction at 530 eV, attributed to the weakening of Zn-O bonds due to the formation of Zn-I bonds and the dispersion of ZnO. Additionally, the doping with ZnO induces an increase in the interlamellar spacing among Bi_2_O_2_ slabs in the BiOI phase, leading to a reduction in the outer-shell electron density of O. The comprehensive XPS analysis validates a dual coupling between the phases in the ZnO and BiOI heterostructure, consistent with previous studies [38,41,42,43,44,45].

## 4. Comparison with Similar Studies

Table 1 displays findings from prior research on the photocatalytic degradation of SCN^−^. Earlier investigations primarily focused on utilizing commercially available TiO_2_ (P25) and various catalyst types to eliminate SCN^−^. In contrast, our study employed a custom-designed heterojunction, incorporating optimally doped BiOI into the ZnO lattice, to successfully eradicate SCN^−^. Furthermore, previously documented data indicate that earlier approaches often required more time, with some instances falling short of achieving 100% efficiency. The efficacy demonstrated by the ZnO-[10%]BiOI configuration affirms its applicability for the efficient degradation of persistent pollutants and its potential in wastewater treatment.

## 5. Conclusions

This study reports the preparation of a ZnO-[10%]BiOI *p-n* heterojunction, and its subsequent characterization and application for the destruction of thiocyanate SCN^−^ under simulated solar radiation. The synthesized heterojunction (5 mg) completely destroyed SCN^−^ in a reaction time of 27 min. When the photocatalyst dosage was varied, it was observed that only 60% SCN^−^ was destroyed at a 2.5 mg photocatalyst dose, while it took only 12 min for 100% ferric thiocyanate elimination with 15 mg of photocatalyst. The influence of the initial concentration of SCN^−^ showed that only 45% of SCN^−^ was destroyed at 100 ppm, 90% at 80 ppm, while 93% destruction efficiency was observed at 60 ppm. The pH of the reaction solution was observed to play a crucial role as inhibited activity was observed at acidic and alkaline pH-values, with acidic pH-values showing a more inhibited performance when compared to alkaline pH-values. The optimum photoactivity was obtained at a neutral pH-value of 7 in the reaction mixture. The experimental reaction data were applied to a pseudo-first-order kinetic model in order to describe the kinetic parameters of the photodestruction process. The consequence of the initial SCN^−^ concentration on the photodestruction efficiency showed that the magnitude of the SCN^−^ destruction is affected by its initial concentration, which depreciates as SCN^−^ concentration increases. At an increased heterojunction dose (15 mg), the rate constant increased from 0.188–0.420 min^−1^, while a rate constant of 0.269 min^−1^ was observed at 10 mg. The quick and efficient destruction of SCN^−^ by the ZnO-[10%]BiOI heterostructure is ascribed to inhibited recombination because of the fabrication of the *p-n* heterojunction composed of ZnO and BiOI. It also led to increased surface area, which provided more active sites for more electrons and holes to participate in the reaction and increased light absorption enabled by the visible light properties of BiOI. The findings from this study could potentially be useful to mining industries for the elimination of cyanide and thiocyanates in mining wastewater.

## Figures and Tables

**Table 1:** Comparison with previous and similar studies.

Catalyst	Synthesis Method	Contaminant	Contaminant Concentration (ppm)	Efficiency (%)	Time (mins)	Reference
S_2_O_8_^2−^ + Fe^3+^	Purchased	SCN^−^	100	100	90	[1]
TiO_2_	Purchased	SCN^−^	100	100	300	[3]
UVC/PS/Fe^3+^	Purchased	SCN^−^	50	99.9	40	[24]
TiO_2_	Purchased	SCN^−^	40	96.5		[27]
Fe(III)/Cr(III) hydroxide	Purchased	SCN^−^	40	33	30	[32]
ZnO-[10%]BiOI	Direct and facile	SCN^−^	40	100	27	This study

## Data Availability

The datasets utilized and/or analyzed during the present study can be obtained from the corresponding author upon request.

## Supplementary Materials

The following supporting information can be downloaded at: https://www.mdpi.com/article/10.3390/ma17153832/s1, Figure S1: EDS spectra and stoichiometery; Figure S2: Absorption spectra and Tauc plots.
