# Supplementary material for: Efficient Photodegradation of Thiocyanate Ions in Mining Wastewater Using a ZnO-BiOI Heterojunction
Source: Materials (Basel). 2024 Aug 2;17(15):3832. doi: 10.3390/ma17153832 (PMC11313042; doi:10.3390/ma17153832)
Supplement: Supplementary file 1 [file materials-17-03832-s001.zip › materials-3075940-supplementary.pdf]

Efficient Photodegradation of Thiocyanate ions in Mining Wastewater Using a ZnO-BiOI Heterojunction

Darlington .C. Ashiegbu [1,*] . David Nkhoesa[1]. Rudolph Erasmus[2] . Herman Johanes Potgieter[1]

[1]. Sustainable and Innovative Metals and Minerals Extraction Technology (SIMMET) Laboratory, School of Chemical and Metallurgical Engineering, University of the Witwatersrand, Private Bag X3, Wits, 2050, South Africa

[2]. School of Physics, University of the Witwatersrand, Private Bag X3, Wits, 2050, South Africa

*Corresponding author: E-mail: darlingtonashiegbu1985@yahoo.com; darlington.ashiegbu@wits.ac.za: 0000-0003-2204-6248 (D.C. Ashiegbu)

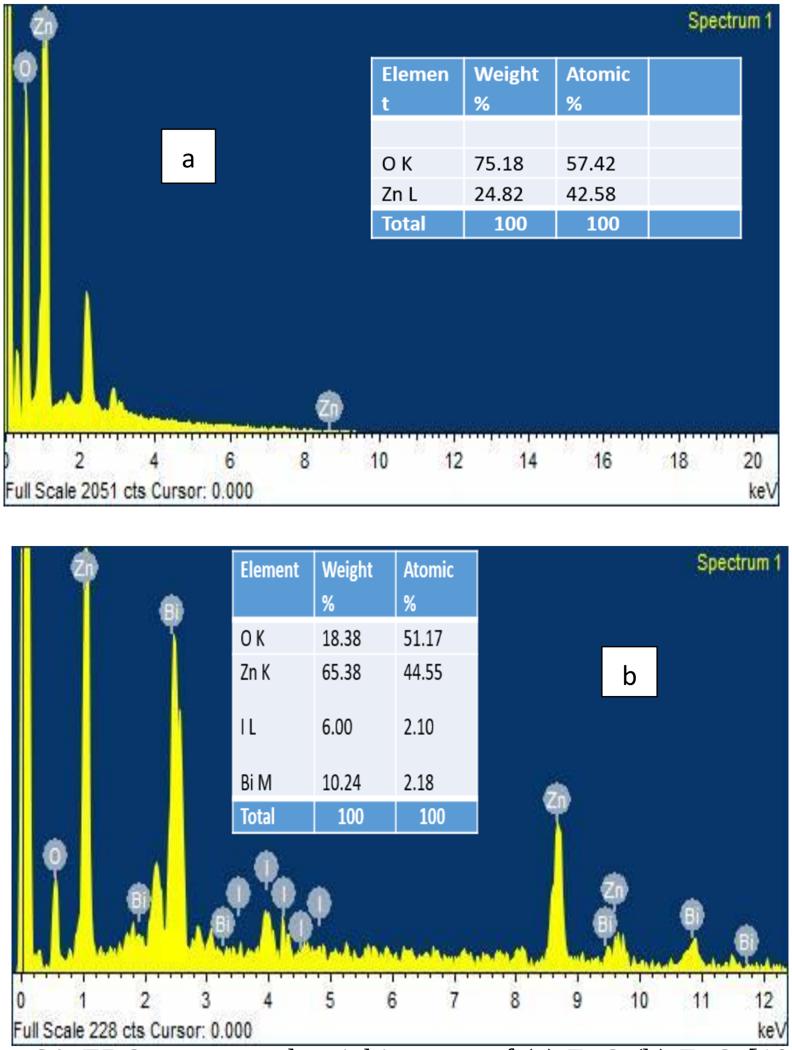

Figure S1. EDS spectra and stoichiometry of (a) ZnO (b) ZnO-[10%]BiOI

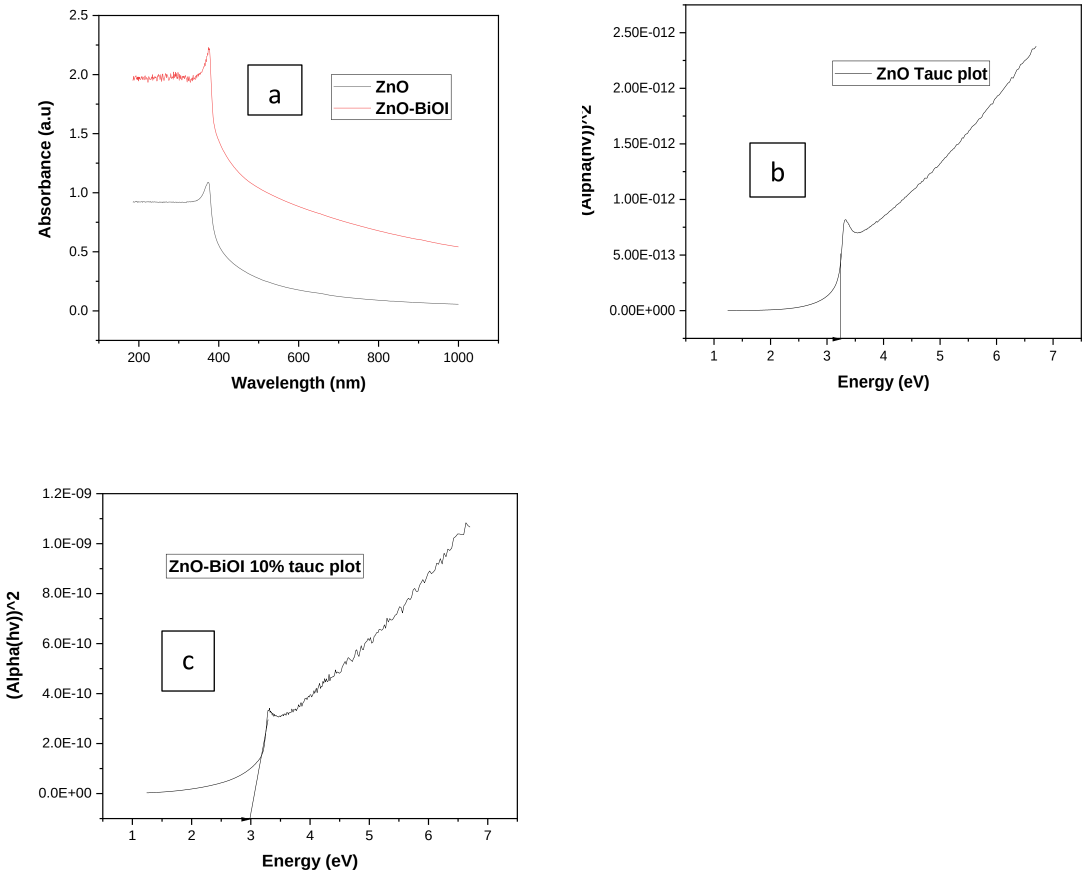

Figure S2. Absorption spectra and Tauc plots of (a) ZnO and ZnO-[10%]BiOI (b) ZnO (c) ZnO-[10%]BiOI
